# Modeling CNS Involvement in Pompe Disease Using Neural Stem Cells Generated from Patient-Derived Induced Pluripotent Stem Cells

**DOI:** 10.3390/cells10010008

**Published:** 2020-12-22

**Authors:** Yu-Shan Cheng, Shu Yang, Junjie Hong, Rong Li, Jeanette Beers, Jizhong Zou, Wenwei Huang, Wei Zheng

**Affiliations:** 1 National Center for Advancing Translational Sciences, National Institutes of Health, Bethesda, MD 20892, USA; shuyangys@gmail.com (S.Y.); hongjjzju@gmail.com (J.H.); ronger33@gmail.com (R.L.); huangwe@mail.nih.gov (W.H.); 2 iPSC Core Facility, National Heart, Lung, and Blood Institute, National Institutes of Health, Bethesda, MD 20892, USA; beersj@mail.nih.gov (J.B.); jizhong.zou@nih.gov (J.Z.)

**Keywords:** Pompe disease, lysosomal storage disease, induced pluripotent stem cells, cell-based disease model, neural stem cells

## Abstract

Pompe disease is a lysosomal storage disorder caused by autosomal recessive mutations in the *acid alpha-glucosidase (GAA)* gene. Acid alpha-glucosidase deficiency leads to abnormal glycogen accumulation in patient cells. Given the increasing evidence of central nervous system (CNS) involvement in classic infantile Pompe disease, we used neural stem cells, differentiated from patient induced pluripotent stem cells, to model the neuronal phenotype of Pompe disease. These Pompe neural stem cells exhibited disease-related phenotypes including glycogen accumulation, increased lysosomal staining, and secondary lipid buildup. These morphological phenotypes in patient neural stem cells provided a tool for drug efficacy evaluation. Two potential therapeutic agents, hydroxypropyl-β-cyclodextrin and δ-tocopherol, were tested along with recombinant human acid alpha-glucosidase (rhGAA) in this cell-based Pompe model. Treatment with rhGAA reduced LysoTracker staining in Pompe neural stem cells, indicating reduced lysosome size. Additionally, treatment of diseased neural stem cells with the combination of hydroxypropyl-β-cyclodextrin and δ-tocopherol significantly reduced the disease phenotypes. These results demonstrated patient-derived Pompe neural stem cells could be used as a model to study disease pathogenesis, to evaluate drug efficacy, and to screen compounds for drug discovery in the context of correcting CNS defects.

## 1. Introduction

Glycogen storage disease type II (OMIM 232300), also known as Pompe disease, is an autosomal recessive metabolic myopathy caused by mutations in the *acid alpha-glucosidase* (*GAA*) gene encoding the lysosomal enzyme GAA. Deficiency of GAA causes glycogen over-accumulation within the lysosomes, resulting in progressive cellular malfunction, cell and tissue damage, and, eventually, organ impairment [1,2]. The primary clinical presentation of Pompe disease is muscle weakness; nonetheless, severe cardiomegaly and cardiomyopathy are crucial features of infantile-onset Pompe disease (IOPD) patients in which cardio-respiratory failure is the leading cause of death [1,3]. Abnormal glycogen storage throughout the central nervous system (CNS), most prominently in spinal ganglia, anterior horns, and motor nuclei brainstem have been reported, which suggests CNS involvement [4,5,6,7]. While detailed brain pathology and its relevance to clinical symptoms are not yet fully understood, recent studies indicate that neuronal abnormalities also contribute to muscle weakness [8]. Enzyme replacement therapy (ERT) with recombinant human GAA (rhGAA) has been approved by the United States Food and Drug Administration (FDA) for Pompe disease. ERT improves survival by correcting cardiac symptoms and partially recovering muscle strength. However, since rhGAA cannot cross the blood–brain barrier, ERT does not ameliorate the neurological symptoms and progressive white matter abnormalities have been observed in rhGAA treated IOPD upon neuroimaging [9,10].

Disease relevant cell-based and animal models are essential approaches for understanding disease pathology and evaluating drug efficacy. Cell-based phenotypic assays are a useful platform for compound screening to identify lead compounds during drug development. Induced pluripotent stem cell (iPSC) technology improves the investigation of disease pathology and the evaluation of drug efficacy, because the models are derived from actual patient samples, leading to more biologically relevant modeling of disease. Indeed, the cardiomyocytes and skeletal muscle cells differentiated from Pompe disease patient iPSCs showed glycogen accumulation, a hallmark of Pompe disease [11,12,13,14,15,16,17]. Furthermore, these models were used to study disease pathogenesis and to evaluate gene therapy.

This work focused on the characterization of Pompe disease phenotypes in neural stem cells (NSCs) differentiated from Pompe disease iPSCs generated from patient fibroblasts. The patient-derived NSCs showed a deficiency of GAA and glycogen buildup. Furthermore, the measurements of two phenotypic assays—LysoTracker staining for enlarged lysosomes and Nile red staining for secondary lipid accumulation—were concordant with patient symptoms. This Pompe disease NSC model was used to evaluate therapeutic responses to δ-tocopherol, hydroxypropyl-beta-cyclodextrin (HPβCD), and recombinant human GAA (rhGAA). δ-tocopherol and HPβCD are two structurally unrelated small molecules that attenuated lysosomal storages in several cell-based models of lysosomal storage disorders mainly through increasing lysosomal exocytosis [18,19,20,21,22,23]. The treatments with δ-tocopherol and HPβCD as well as the rhGAA ameliorated disease phenotypes in Pompe disease NCSs. The results demonstrated that the Pompe disease patient-derived NSCs successfully modeled CNS involvement in Pompe disease. This tool can be used for compound screening and validating new therapeutics to treat Pompe disease.

## 2. Materials and Methods

### 2.1. Materials

CTS CELLstart substrate (A1014201), CytoTune-iPS 2.0 Sendai reprogramming kit (A16517), Hoechst 33342 trihydrochloride (H3570), LysoTracker red DND-99 (L7528), PSC neural induction medium (A1647801), Nile red (N-1142), StemPro NSC SFM (A1050901), and StemFlex medium (A3349401) were purchased from Thermo Fisher Scientific (Waltham, MA, USA). ROCK inhibitor Y-27632 (1284) was purchased from Tocris Bioscience (Ellisville, MO, USA). Matrigel (354277) was obtained from Corning (Corning, New York, NY, USA). Accutase (07920), and mTeSR1 medium (85850) were purchased from StemCell Technologies (Vancouver, BC, Canada). Delta-tocopherol was purchased from Sigma-Aldrich (St. Louis, MO, USA) and purified by HPLC to a purity greater than 99% [20]. Dermal fibroblast lines (GM20122, GM00248, and GM00338) were obtained from the NIGMS Human Genetic Cell Repository at the Coriell Institute for Medical Research.

### 2.2. Induced Pluripotent Stem Cell Generation and Culture

Induced pluripotent stem cells (iPSCs) were generated with a CytoTune-iPS 2.0 Sendai reprogramming kit following a previously described protocol [24,25]. Briefly, each fibroblast line was transduced and cultured in one well of a 48-well plate. Cells were maintained in reprogramming medium 1, and the medium was replaced every other day. On day 4, cells were replated onto a Matrigel pre-coated 48-well plate with different cell numbers and cultured in reprogramming medium 2. Medium was replaced every other day. At day 20, the medium was switched to Essential 8 medium, and cells were fed every day until day 25. For the first three passages, selected colonies were cultured and expanded in Essential 8 medium. iPSCs were further cultured in either StemFlex or mTeSR1 medium on Matrigel pre-coated six-well plates. Passaged every three to five days, iPSCs were loosened from the plates with 0.5 mM ethylenediaminetetraacetic acid (EDTA) upon reaching 70% to 80% confluence.

### 2.3. Neural Stem Cell Differentiation and Maintenance

NSC lines were generated using the PSC Neural Induction Medium as described in the manufacturer’s manual (A1647801, Thermo Fisher Scientific, Waltham, MA, USA). Briefly, iPSCs cultured in StemFlex were split—a small portion of the cells was separated out for cell counting after further dissociation—and the remaining cell clumps were seeded into six-well plates at a density of 3 × 10^5^ cells/well. Cells were switched into PSC Neural Induction Medium after 24 h and fed every other day. After day 4, the medium was changed every day. At day 7, primitive NSCs were harvested with Accutase and seeded in Matrigel pre-coated T75 flasks in NSC expansion medium. Cells were fed every day and reached confluence after five days. ROCK inhibitor Y27632 (5 µM) was added to the NSC expansion medium at the time of plating before the fourth passage.

### 2.4. Immunocytochemistry (ICC) Staining

Cells were fixed in 4% paraformaldehyde for 15 min, rinsed with Dulbecco’s phosphate buffered saline (DPBS), and permeabilized with 0.5% Triton X-100 for 10 min at room temperature prior to immunostaining. Nonspecific sites were first blocked with cell staining buffer (BioLegend, San Diego, CA, USA) for 1 h at room temperature. Cells were then incubated with primary antibodies (Table S1) overnight at 4 °C. After rinsing three times with DPBS, cells were stained with corresponding secondary antibodies for 1 h at room temperature (Table S1). Cells were rinsed once, and the nuclei were stained with Hoechst 33342 for 15 min at 37 °C. After another two rinses, followed by filling with DPBS, cells were visualized using an IN Cell Analyzer 2500 imaging system (GE Healthcare, Port Washington, NY, USA).

### 2.5. LysoTracker and Nile Red Staining

NSCs were seeded on 96-well plates pre-coated with CTS CELLstart substrate at a density of 3000 to 5000 cells per well. The medium was changed to StemPro NSC SFM medium supplemented with the desired treatment and 3% fetal bovine serum (FBS) on the next day. After a 72 h incubation period, cells were first washed with warm StemPro NSC SFM medium and switched into medium containing 50 nM LysoTracker red or 1 µM of Nile red dye. For LysoTracker staining, cells were incubated for 30 min at 37 °C; for Nile red staining, cells were incubated for 10 min at 37 °C. After incubation, cells were fixed with 4% paraformaldehyde, and the nuclei were stained with Hoechst 33342 for 15 min at 37 °C. After rinsing with DPBS, cells were imaged on an IN Cell Analyzer 2500 imaging system (GE Health) at 20X magnification.

### 2.6. Western Blot Analysis

NSCs harvested by Accutase were pelleted and lysed in cold RIPA lysis buffer containing protease inhibitors (cOmplete mini tablets, 4693159001, Sigma-Aldrich, St. Louis, MO, USA. Protein concentrations were determined using the BCA protein assay kit (Pierce). Anti-GAA (1:400, EPR4716(2), Abcam, Cambridge, MA, USA) or anti-GAPDH (1:1000, sc-25778, Santa Cruz Biotechnology, Dallas, TX, USA) antibodies were used.

### 2.7. Glycogen Quantification

NSCs were harvested using Accutase and washed once with cold DPBS. Cell pellets were resuspended in cold water and separated into two portions. One portion was used to measure the protein concentration via a bicinchoninic acid (BCA) assay after lysis in cold RIPA buffer. The other portion was used to quantify the total amount of cellular glycogen with the glycogen colorimetric/fluorometric assay kit (K646, BioVision, Milpitas, CA, USA).

### 2.8. Fluorometric GAA Enzyme Assay

NSCs were assayed for GAA enzyme activity. Cells were dissociated using Accutase and incubated in a lysis buffer containing 50 mM Tris pH 7.5, 100 mM NaCl, 1% Triton X-100, and protease inhibitors (cOmplete mini tablets, 4693159001, Sigma-Aldrich, St. Louis, MO, USA) for 30 min on ice. After centrifugation, supernatant was collected for assays. Protein concentrations were measured with a BCA assay. The GAA activity assay was adapted from [26]. Briefly, 15 µL of the cell lysates with 10 µg total proteins were incubated with 40 µL of 2.2 mM fluorogenic substrate 4-methylumbelliferyl-α-D-glucopyranoside (4MU, Sigma-Aldrich, St. Louis, MO, USA) in 200 mM sodium acetate buffer, pH 3.8, plus 5 µL of 960 µM acarbose (Sigma-Aldrich, St. Louis, MO, USA) in ddH_2_O at 37 °C for 1 h in a 96-well plate. Reactions were terminated by adding 200 µL of 150 mM EDTA, pH 11.4. Fluorescence was measured at 360 nm (excitation) and 465 nm (emission) on a CLARIOstar microplate reader (BMG LABTECH).

### 2.9. Recombinant Human GAA Treatment

Recombinant human GAA (rhGAA) enzyme was obtained from the residual solution after the clinical infusions of Lumizyme (Sanofi Genzyme, Cambridge, MA, USA). The enzyme solution was stored in 30% glycerol at −80 °C. For Nile red or LysoTracker staining, NSCs were seeded on black, clear bottom, 96-well plates pre-coated with CTS CELLstart substrate at a density of 3000 to 5000 cells per well. The medium was changed to StemPro NSC SFM medium supplemented with rhGAA and 3% FBS on the next day. Recombinant human GAA was removed after 12 h. After a 72-h incubation, Nile red or LysoTracker staining was performed. For GAA assay and glycogen quantification, NSCs were seeded on 12-well plates pre-coated with Matrigel at a density of 10^5^ to 2 × 10^5^ cells per well, followed by the same treatment procedure. After a 72-h incubation, cells were washed twice with DPBS and harvested using Accutase. After another two cold DPBS washes, cells were examined with either GAA or glycogen assays as described earlier.

### 2.10. Treatment with HPβCD and δ-Tocopherol

Cells were seeded on 96-well plates pre-coated with CTS CELLstart substrate. The medium was changed to StemPro NSC SFM medium supplemented with the desired treatment and 3% FBS on the next day. After a 72-h incubation, cells were examined with either LysoTracker or Nile red staining as described earlier.

### 2.11. Data Analysis and Statistics

Images were analyzed with an IN Cell Analyzer Workstation using a multi-target analysis. Segmentation was conducted for nuclei, cytoplasma, and organelle structures. Statistical significance was determined with *p*-values calculated from paired, one-tailed Student’s *t* test using GraphPad Prism (version 8.0.1); error bars represent the standard error of the mean (SEM). Significant scores were present as *p* values where * indicates *p* < 0.05, ** indicates *p* < 0.01, and *** indicated *p* < 0.001.

## 3. Results

### 3.1. Generation and Characterization of Pompe Disease iPSCs from Pompe Patient Fibroblasts

Three Pompe disease patient dermal fibroblast lines obtained from Coriell Cell Repositories (GM20122, GM00248, and GM00338) were reprogrammed into three iPSC lines: NCATS-CL9316, NCATS-CL9317, and NCATS-CL9318. All Pompe disease cell lines carried the homozygous nonsense mutation p. R845X in GAA according to the gene mutation data from Coriell. The R854X mutation is prevalent in patients of African or African American descent with an allele frequency of 0.2% [27]. These iPSC lines showed a typical iPSC morphology and expressed pluripotency markers as shown in Figure S1A, including SOX2, Nanog, and Oct4 in the nuclei, and Tra-1-60 and SSEA4 on the plasma membrane in the NCATS-CL9316 and NCATS-CL9318 iPSC lines, while the characterization of iPSC line NCATS-CL9317 (formerly named as TRNDi007-B) was reported in detail previously [28]. Cell line identities were verified through a short tandem repeat (STR) DNA analysis (data upon request) and all iPSCs maintained a normal karyotype (Figure S1B). The three Pompe disease iPSC lines generated from Pompe disease patient samples also showed a normal growth rate compared to wild type iPSC controls (iPSC HT268A, derived from health donor fibroblasts GM05659 [29]).

### 3.2. NSC Differentiation and Characterization

**Figure 1:**
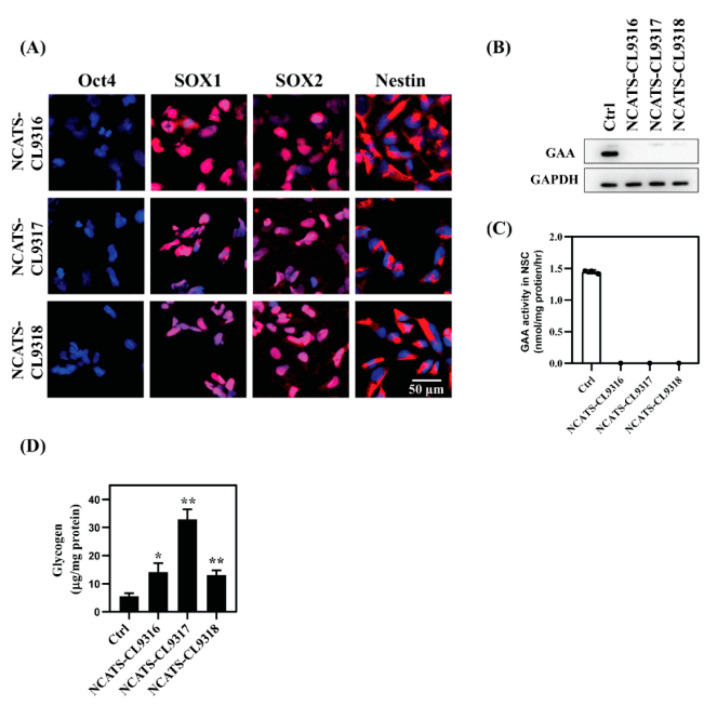
Characterization of Pompe disease patient-derived neural stem cells (NSCs) (**A**) Immunofluorescent staining of Pompe disease NSCs showing positive staining of neural markers (SOX1, SOX2, and Nestin) and negative staining for the pluripotency marker, Oct4. Blue staining indicates nuclear (Hoechst) staining. (**B**) Western blot analysis of GAA protein levels in control (Ctrl) versus Pompe disease NSCs. (**C**) GAA activity assays using 4-MU substrate in control and patient NSCs. (**D**) Total amount of cellular glycogen of control and patient NSCs. Error bars represent SEM of three independent experiments (* *p* < 0.05 and ** *p* < 0.01 by *t*-test).

To test the efficacy of therapeutic agents on neuronal cells, Pompe disease iPSCs were subsequently differentiated into NSCs, which is relatively easy to be generated and this differentiation is highly reproducible. NSC markers, including SOX1 and SOX2 in the nuclei and Nestin in the plasma membrane of these cells were observed with immunocytochemistry staining (Figure 1A). We then examined disease phenotypes in three patient-derived NSC lines compared to a control (Ctrl) NSC line (Fibroblast Coriell ID: GM05659, iPSC ID:HT268A) [29]. No GAA enzyme was detected by western blotting in the Pompe disease NSCs (Figure 1B); the same was also observed in the patient-derived fibroblasts, the source cells from Coriell Cell Repository [30]. All three Pompe disease cell lines harbored a homozygous nonsense mutation p. R845X in GAA, resulting in no detectable mRNA for GAA and essentially no GAA activity in patient fibroblasts [31]. Next, GAA enzyme activity was examined in these NSCs. All Pompe disease NSCs showed no GAA activity, suggesting the GAA deficiency nature was preserved in the Pompe disease NSCs (Figure 1C).

Because the deficiency of GAA leads to glycogen build-up in patient cells, we investigated glycogen levels in the Pompe disease NSCs using a biochemical assay [26] and found that total intracellular glycogen increased in all three Pompe disease NSC lines compared with the control cells (Figure 1D). The NCATS-CL9316 and NCATS-CL9318 NSCs exhibited a 2.3- to 2.5-fold glycogen increase, and NCATS-CL9317 NSCs showed a 6-fold increase. Together, the patient NSCs had negligible GAA activity accompanied with the accumulation of glycogen. The replication of Pompe disease patient phenotypes in these iPSC differentiated NSCs indicated that these cells could serve as a cell-based disease model to study Pompe disease pathophysiology.

### 3.3. Enlarged Lysosomes and Neutral Lipid Increase in Pompe Disease NSCs

**Figure 2:**
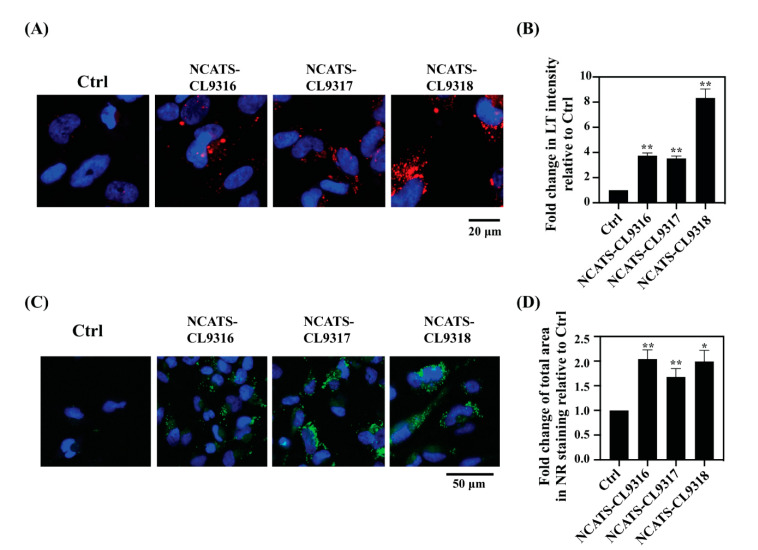
Pompe disease NSCs present increased lysosomal staining and lipid droplet accumulation. (**A**) Representative images for LysoTracker staining (red) in NSCs. Nuclei are stained with Hoechst (blue); merged images are shown. (**B**) Bar diagram presents the quantitation of LysoTracker intensity in disease lines relative to the control (Ctrl) cells. Error bars represent SEM of three independent experiments (** *p* < 0.01 by *t*-test). (**C**) Representative images for Nile red staining (green) of NSCs. Nuclei are stained with Hoechst (blue); merged images are shown. (**D**) Bar diagram presents the quantitation of total area of the stained puncta in disease lines relative to the control cells. Error bars represent SEM of three independent experiments (* *p* < 0.05 and ** *p* < 0.01 by *t*-test).

To characterize disease phenotypes in the patient-derived cells, we used LysoTracker dye staining to measure enlarged lysosomes and Nile red dye staining to determine secondary lipid accumulation. LysoTracker, an acidotropic reagent, is used to visualize acidic vesicles in cells, and its staining increases in patient cells of many lysosomal storage diseases [32]. Total fluorescent intensity of LysoTracker staining increased in all Pompe disease NSCs after the addition of FBS containing lipids to the cells (Figure 2A). The NCATS-CL9316 and NCATS-CL9317 NSCs showed 3.5- to 3.7-fold increases in LysoTracker staining, while the NCATS-CL9318 NSCs showed an 8-fold increase, compared with control NSCs (Figure 2B), indicating enlarged lysosomes in the Pompe disease patient-derived NSCs. As previously reported that secondary lipid accumulation occurred in the Pompe disease patients [33,34], we found that all Pompe disease NSCs showed 1.7- to 2.0-fold increases of the Nile red dye staining (Figure 2C,D), indicating the accumulation of nonpolar lipids. The increases of LysoTracker and Nile red staining evidenced the phenotypical changes of enlarged lysosomes and the secondary accumulation of lipids in Pompe disease NSCs.

### 3.4. Recombinant Human GAA Decreased the Phenotypes in Pompe Disease NSCs

**Figure 3:**
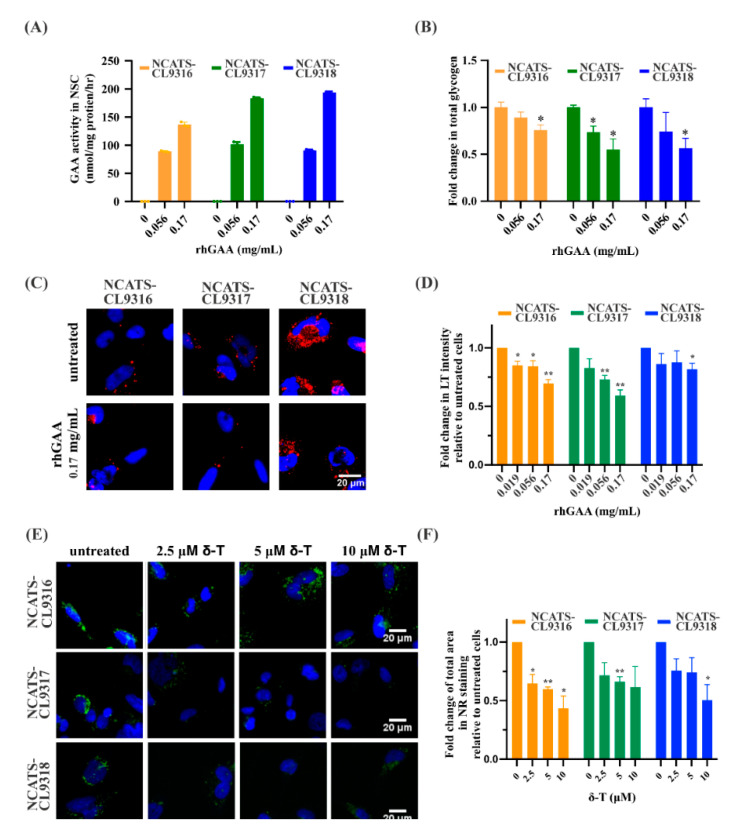
Phenotypic effects of rhGAA and δ-tocopherol in Pompe disease NSCs. (**A**) Bar graph presents GAA activities in rhGAA treated NSCs. (**B**) Bar graph presents fold change of total amount of cellular glycogen in disease NSCs with rhGAA treatment. (* *p* < 0.05 by *t*-test; errors bars are derived from the SEM of three independent experiments). (**C**) Representative images of LysoTracker staining (red) in rhGAA treated NSCs. (**D**) Bar graph presents the quantitation of LysoTracker (LT) staining after rhGAA treatment; blue (Hoechst) staining indicates nuclei (* *p* < 0.05 and ** *p* < 0.01 by *t*-test; errors bars are derived from the SEM of three independent experiments). (**E**) Representative images of Nile red staining (green) of δ-tocopherol (δ-T) treated NSCs. (**F**) Bar graph presents the quantitation of Nile red (NR) staining after δ-tocopherol treatment. Error bars represent SEM of three independent experiments (* *p* < 0.05 and ** *p* < 0.01 by *t*-test).

To determine the effect of enzyme replacement therapy in the Pompe disease NSCs, we applied recombinant human GAA protein (rhGAA) to these patient-derived cells. After a 12-h treatment followed by a three-day incubation, GAA activity significantly increased (Figure 3A). In a dose-dependent manner, total intracellular glycogen and LysoTracker intensity in all patient lines decreased with higher rhGAA concentrations (Figure 3B–D). At 0.17 mg/mL rhGAA, a 30% reduction in LysoTracker staining was observed for the NCATS-CL9316 NSCs, 40% for the NCATS-CL9317 NSCs, and 20% for the NCATS-CL9318 NSCs (Figure 3C,D). However, we did not observe significant effects of rhGAA on the Nile red staining (Figure S2A), indicating a limited effect on the secondary lipid accumulation in these Pompe disease cells.

### 3.5. Delta-Tocopherol Ameliorated Lipid Accumulation in Pompe Disease NSCs

Next, we examined the effects of two compounds, δ-tocopherol and HPβCD. These compounds reduce lipid accumulation for several lysosomal storage diseases [20,21]. After δ-tocopherol treatment, the Nile red staining was dose-dependently reduced in all Pompe disease NSCs. Compared to untreated patient cells, 10 µM δ-tocopherol resulted in a 57% reduction of Nile red dye staining in the NCATS-CL9316 NSCs, 39% in the NCATS-CL9317 NSCs, and 50% in the NCATS-CL9318 NSCs (Figure 3E,F). LysoTracker staining, on the other hand, did not change in any of the three Pompe disease NSCs after being treated with δ-tocopherol (Figure S2B). For HPβCD, we did not observe significant effects on either LysoTracker staining or Nile red staining in Pompe disease NSCs after the treatment. Interestingly, δ-tocopherol reduced secondary lipid accumulation without significantly decreasing the size of the enlarged lysosomes. This is the opposite of the observed effect of rhGAA treatment in Pompe disease NSCs, suggesting that δ-tocopherol might not reduce lysosomal glycogen accumulation, although it reduced the secondary lipid accumulation.

### 3.6. Combination of δ-Tocopherol and HPβCD Reduced Disease Phenotypes in Pompe Disease NSCs

**Figure 4:**
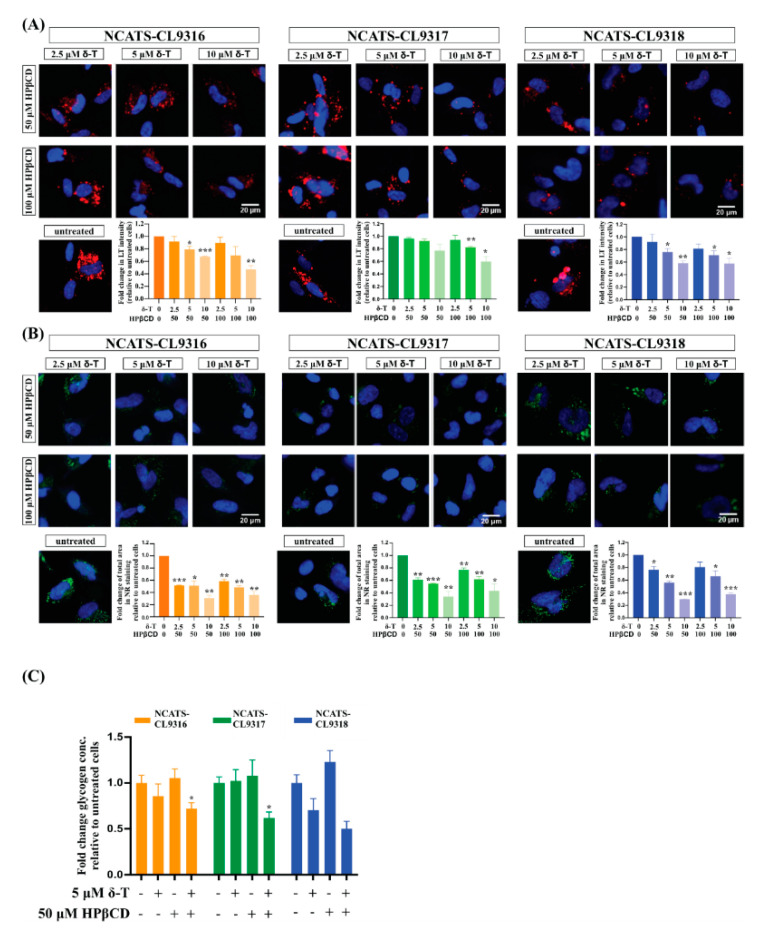
Therapeutic effects of the δ-tocopherol and HPβCD combination in Pompe disease NSCs. (**A**) Representative images of LysoTracker (LT) staining (red) of combination treated NSCs. Bar graph presents the quantitation of LysoTracker staining after the combination treatment. Error bars represent SEM of three independent experiments (* *p* < 0.05, ** *p* < 0.01, and *** *p* < 0. 001 by *t*-test). (**B**) Representative images of Nile red (NR) staining (green) of combination treated NSCs. Bar graph presents the quantitation of Nile red staining after combination treatment. Error bars represent SEM of three independent experiments (* *p* < 0.05, ** *p* < 0.01, and *** *p* < 0.001 by *t*-test). (**C**) Changes of total amount of cellular glycogen in NSCs after treated with δ-tocopherol (δ-T), HPβCD and their combination. Error bars represent SEM of three independent experiments (* *p* < 0.05 by *t*-test).

Previously, we found δ-tocopherol and HPβCD produced an additive or synergistic therapeutic effect on reducing disease phenotypes of patient-derived cells in several lysosomal storage diseases [18,21,22]. Therefore, we treated Pompe disease NSCs with δ-tocopherol plus HPβCD. In the presence of HPβCD, Nile red staining significantly decreased after patient-derived NSCs were treated with low concentrations of δ-tocopherol (Figure 4A,B). The reduction of Nile red staining was further enhanced with higher concentrations of δ-tocopherol in combination with HPβCD compared to those treated only with δ-tocopherol, indicating an additive or synergistic effect on the decrease of accumulated neutral lipids in the Pompe disease NSCs. In addition, the combination therapy significantly reduced LysoTracker staining in the Pompe disease NSCs although no reduction was observed with the solo treatment of δ-tocopherol or HPβCD. We also observed the combination of δ-tocopherol with HPβCD reduced glycogen accumulation in the Pompe disease NSCs (Figure 4C) while the effects were limited and in only two of the three disease lines. Together, these results suggest combining δ-tocopherol and HPβCD effectively ameliorated the disease phenotypes of glycogen accumulation, secondary lipid accumulation, and abnormal lysosomal size in Pompe disease NSCs.

## 4. Discussion

Besides the well-known cardiac and skeletal abnormalities caused by glycogen accumulation, clinical and animal data indicate the central nervous system (CNS) contributes to the myopathy of Pompe disease [4,7]. However, the neuropathology of Pompe disease is still not fully characterized; the available Pompe disease mice models cannot fully recapitulate the human clinical phenotypes. Advancements in iPSC technology offer an alternative approach to studying the disease phenotype and pathology employing patient-derived neuronal cells. iPSC-derived NSCs have been applied to study a wide variety of neurological diseases, including several lysosomal disorder diseases that recapitulate known disease phenotypes and mediate the discovery of new disease physiology [19,22,29,35,36,37,38].

In this study, we generated iPSCs from Pompe disease patients and differentiated them into NSCs for use as a disease model system to investigate Pompe disease. Phenotypic markers of Pompe disease, including glycogen accumulation, lysosome enlargement, and secondary lipid accumulation, were all found in the patient-derived NSCs. Furthermore, the proliferative nature of NSCs allows for the generation of large numbers; hence, the Pompe disease NSCs can be used as a cell-based disease model with potential for high-throughput screening in drug discovery.

As a proof of concept (to show potential applications of this model in drug discovery with high throughput screening), we tested rhGAA, δ-tocopherol, and HPβCD in this Pompe disease NSC model in a 96-well plate format. δ-tocopherol is one of eight components in vitamin E, of which antioxidant and anti-inflammatory activity are most commonly considered biological functions [39]. In addition, its cancer preventive activity has been demonstrated in the animal models of breast, colon, and lung cancer [40,41,42]. δ-tocopherol can also inhibit prostate cancer cell growth [43]. HPβCD is a cyclic oligosaccharide composed of seven glycosidic residues. It is used as an excipient in the drug formulation to increase the oral bioavailability for lipophilic drugs [44]. In addition to the mentioned application, δ-tocopherol and HPβCD were reported to reduce the disease phenotypes of lysosomal storage diseases in *in vitro* models [18,19,20,21,22,29]. The therapeutic potential of HPβCD was also confirmed in animal models [45,46,47,48,49]. Mechanistic studies in Niemann-Pick type C disease cells suggest the therapeutic effects of both δ-tocopherol and HPβCD are associated with the increase of lysosomal exocytosis [20,23]. The ERT therapy with rhGAA resulted in an increase of GAA activity, a decrease of total cellular glycogen level, and a partial reduction of LysoTracker staining in patient-derived NSCs. We also found δ-tocopherol significantly reduced Nile red dye staining, an indication of more normal levels of secondary lipid accumulation, but it did not affect the increased, abnormal LysoTracker staining in patient-derived cells. In combination, δ-tocopherol and HPβCD, significantly reduced both LysoTracker staining and Nile red staining, indicating alleviations of both enlarged lysosomes and secondary lipid accumulation in Pompe disease NSCs. These results demonstrated iPSCs derived Pompe disease NSCs can be a useful tool to evaluate drug efficacy as well as for compound screening in a high throughput format.

The different effects of rhGAA and δ-tocopherol on LysoTracker staining and the Nile red staining could be caused by the different mechanisms of action of these two drugs. ERT corrects the GAA deficiency resulting in the degradation of abnormally-accumulated glycogen in lysosomes, which, in turn, is evidenced by a decrease in LysoTracker staining compared to the untreated disease cells. However, only a modest decrease (20% to 40% reduction) of LysoTracker staining was observed in the ERT-treated patient-derived cells. As a possible explanation, the accumulation of secondary lipids in diseased cells is less sensitive to rhGAA treatment under the current experimental conditions. This result is in line with the observation that ERT does not reverse, but rather attenuates disease progression in patients [50,51,52,53,54]. Autophagic defects persisted in patients and mice even after long term ERT [55,56]. Noteworthily, a newly developed ERT (ATB200/AT2221) markedly reduces autophagic buildup in the muscle of *Gaa*-KO mice [57]. This result suggests that high doses of ERT may reverse the autophagic defects as ATB200 has higher M6P, which improve its uptake in muscle compared to the currently used Alglucosidase alfa. Therefore, further study is needed to elucidate the hypothesis of autophagic buildup in disease NSCs.

Delta-tocopherol and its combination with HPβCD showed stronger effects in reducing secondary lipid accumulation than lysotracker staining in Pompe disease NSCs. This could attribute to a similar mechanism of action in reducing the abnormal over-accumulation of cholesterol and lipids reported previously in other lysosomal disease cells [20]. The reduction of LysoTracker staining in the combination treatment—δ-tocopherol and HPβCD—indicated secondary lipid accumulation also contributed to the lysosome enlargement in the patient-derived disease cells. These results suggested the combination of δ-tocopherol and HPβCD merit further study as a potential therapy for Pompe disease in patients. Further mechanistic study of the combination may help to expand the application of this combination and identify new targets to treat lysosomal storage diseases.

The present study represents the first attempt to study Pompe disease using iPSC derived NSCs. A limitation of this study is that we only focused on NSCs, excluding other cell types, with only three patient-derived lines (all with the same mutation) and one control line. Samples carrying the same disease related mutation but with different genetic background gives different degrees of phenotypes, as shown in this study. Therefore, examining Pompe disease NSCs with different mutations as well as additional control lines can further clarify and extend the application of this model. While we were preparing the manuscript, a paper using Pompe disease iPSC derived neurons to test small molecules was published; the authors reported glycogen accumulation in the disease cells [58]. Their findings support the idea of using Pompe disease iPSC derived neurons as a model system to evaluate Pompe disease therapies for effects on the nervous system. However, both our study and the one published in 2019 share the same limitation: only morphological and biochemical alterations were assessed. Further neuronal activity assays are required to better understand the neuropathology and thus guide drug discovery.

In conclusion, we successfully established an in vitro NSC model using patient-derived iPSCs and identified two disease-related phenotypes which are suitable for high-throughput screening applications. The therapeutic effect of combining δ-tocopherol and HPβCD on Pompe disease should be further studied.

## Supplementary Materials

The following are available online at https://www.mdpi.com/2073-4409/10/1/8/s1. Figure S1: Characterization of Pompe disease patient derived iPSCs, Figure S2: Response to rhGAA and δ-tocopherol treatment. Table S1: Antibodies used in immunocytochemical staining.
