# Supplementary material for: Modeling CNS Involvement in Pompe Disease Using Neural Stem Cells Generated from Patient-Derived Induced Pluripotent Stem Cells
Source: Cells. 2020 Dec 22;10(1):8. doi: 10.3390/cells10010008 (PMC7822217; doi:10.3390/cells10010008)
Supplement: Supplementary file 1 [file cells-10-00008-s001.zip › Fig S2.pdf]

**Fig. S2**

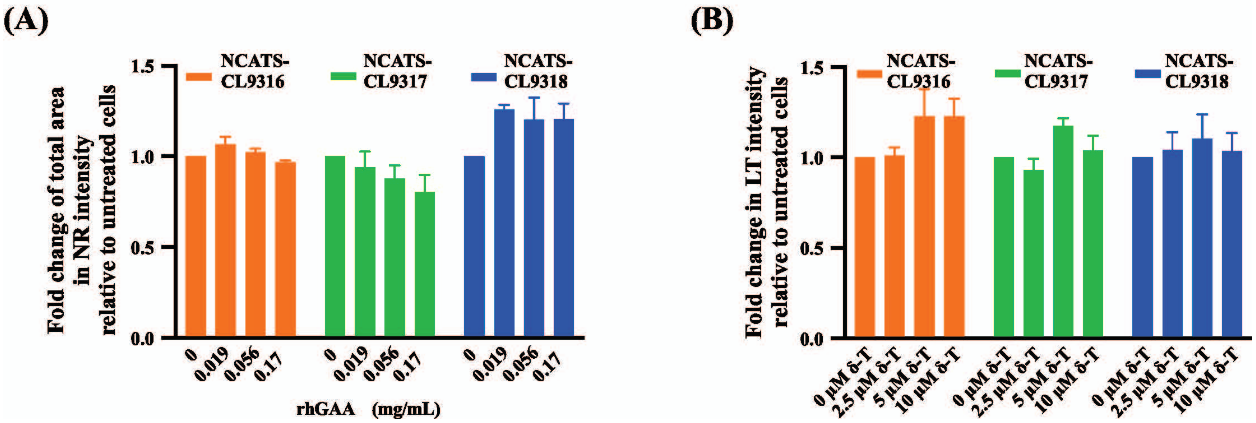


Fig. S2 Response to rhGAA and δ-tocopherol treatment. (A) Bar graph presents the quantitation of Nile red (NR) staining after rhGAA treatment. (B) Bar graph presents the quantitation of Lysotracker (LT) staining after δ-tocopherol (δ-T) treatment.
