# Supplementary material for: Modeling CNS Involvement in Pompe Disease Using Neural Stem Cells Generated from Patient-Derived Induced Pluripotent Stem Cells
Source: Cells. 2020 Dec 22;10(1):8. doi: 10.3390/cells10010008 (PMC7822217; doi:10.3390/cells10010008)
Supplement: Supplementary file 1 [file cells-10-00008-s001.zip › FigS1.pdf]

Fig. S1

(A)

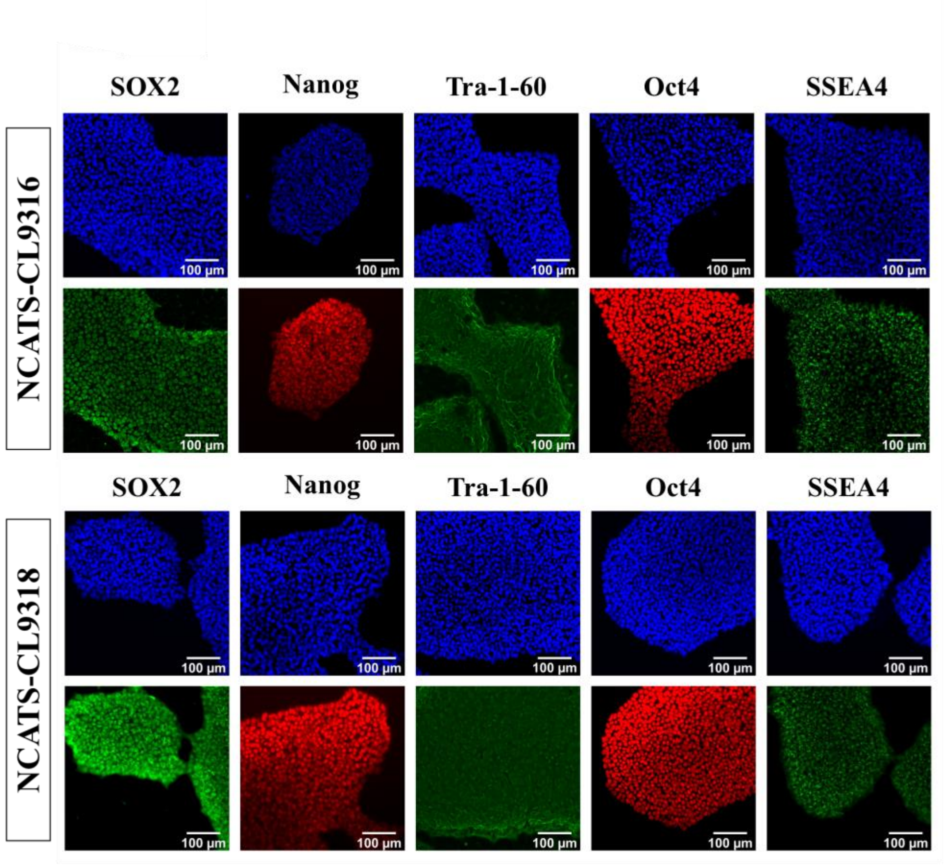

(B)

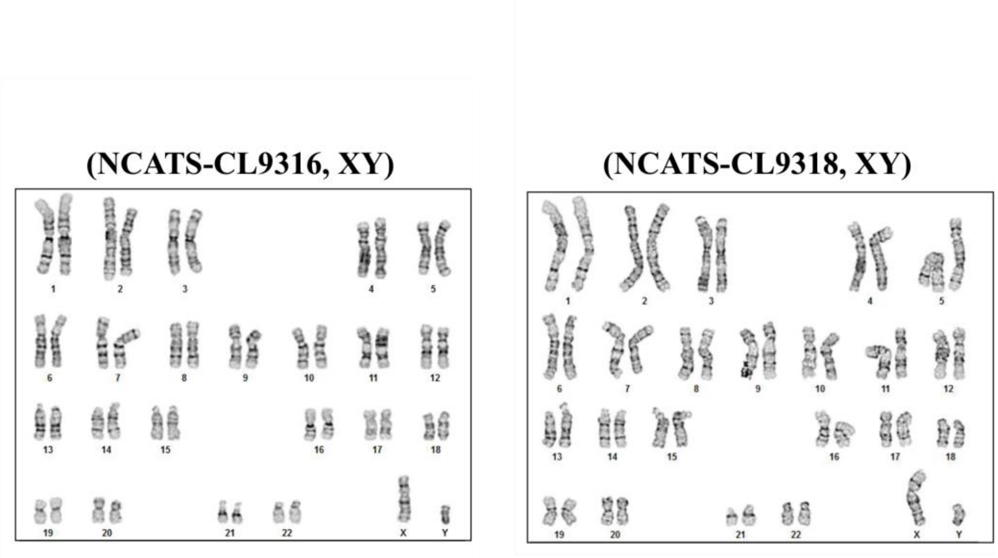

Fig. S1 Characterization of PD patient derived iPSCs. (A) Immunofluorescence images demonstrate the expression of pluripotent markers in iPSC lines NCATS-CL9316 and NCATS-CL9318. (B) Karyotype analysis for iPSC lines NCATS-CL9316 and NCATS-CL9318, indicating normal karyotypes.
