# Supplementary material for: Modeling CNS Involvement in Pompe Disease Using Neural Stem Cells Generated from Patient-Derived Induced Pluripotent Stem Cells
Source: Cells. 2020 Dec 22;10(1):8. doi: 10.3390/cells10010008 (PMC7822217; doi:10.3390/cells10010008)
Supplement: Supplementary file 1 [file cells-10-00008-s001.zip › supplemental table.pdf]

Table S1: Antibodies used in immunocytochemical staining

	**Antibody**	**Dilution**	**Company Catalog Number and RRID**
Pluripotency marker	Mouse anti-SOX2	1:50	R&D Systems, Cat# MAB2018<br>RRID: AB_358009
Pluripotency marker	Mouse anti-SSEA4	1:1000	Cell Signaling Technology, Cat# 4755<br>RRID: AB_1264259
Pluripotency marker	Rabbit anti-NANOG	1:400	Cell Signaling Technology Cat# 4903<br>RRID: AB_10559205
Pluripotency marker	Mouse anti-TRA-1-60	1:500	Cell Signaling Technology Cat# 4746<br>RRID: AB_2119059
Pluripotency marker	Rabbit anti-OCT4A	1:400	Thermo Fisher Cat# A13998<br>RRID: AB_2534182
Neural stem cell marker	Goat anti-SOX1	1:20	R&D Systems, Cat# AF3369<br>RRID: AB_2239879
Neural stem cell marker	Mouse anti-Nestin	1:200	BD Biosciences Cat# 611658<br>RRID: AB_399176
Secondary antibody	Donkey anti-mouse IgG (Alexa Fluor 488)	1:400	Thermo Fisher, Cat# A21202<br>RRID: AB_141607
Secondary antibody	Donkey anti-Rabbit IgG (Alexa Fluor 594)	1:400	Thermo Fisher, Cat# A21207,<br>RRID: AB_141637
Secondary antibody	Donkey anti-Goat IgG (Alexa Fluor 488)	1:400	Thermo Fisher, Cat# A11055,<br>RRID: AB_2534102
